# Cooperative Monocular-Based SLAM for Multi-UAV Systems in GPS-Denied Environments †

**DOI:** 10.3390/s18051351

**Published:** 2018-04-26

**Authors:** Juan-Carlos Trujillo, Rodrigo Munguia, Edmundo Guerra, Antoni Grau

**Affiliations:** 1 Department of Computer Science, CUCEI, University of Guadalajara, Guadalajara 44430, Mexico; juancarlos_max@hotmail.com(J.-C.T.); rodrigo.munguia@upc.edu (R.M.); 2 Department of Automatic Control, Technical University of Catalonia UPC, 08034 Barcelona, Spain; edmundo.guerra@upc.edu

**Keywords:** state estimation, unmanned aerial vehicle, monocular vision, localization, mapping, observability, cooperative

## Abstract

This work presents a cooperative monocular-based SLAM approach for multi-UAV systems that can operate in GPS-denied environments. The main contribution of the work is to show that, using visual information obtained from monocular cameras mounted onboard aerial vehicles flying in formation, the observability properties of the whole system are improved. This fact is especially notorious when compared with other related visual SLAM configurations. In order to improve the observability properties, some measurements of the relative distance between the UAVs are included in the system. These relative distances are also obtained from visual information. The proposed approach is theoretically validated by means of a nonlinear observability analysis. Furthermore, an extensive set of computer simulations is presented in order to validate the proposed approach. The numerical simulation results show that the proposed system is able to provide a good position and orientation estimation of the aerial vehicles flying in formation.

## 1. Introduction

Nowadays, unmanned aerial vehicles (UAVs) have received great attention from the robotics research community. In this case, one of the main objectives has been the improvement of the autonomy of these systems. In particular, the multi-rotor aerial systems allow great versatility of movements, making this kind of aerial platform very useful for a great variety of applications. Altogether with the recent advances in computational processing, computer vision has become an important tool in order to improve the autonomy of robotics systems. Cameras are well adapted for embedded systems because they are inexpensive, lightweight and power-saving. For instance, applications of surveillance [1], tracking and rescue [2], among others, seem to be feasible for aerial robots equipped with onboard cameras.

A fundamental requirement in order to improve the autonomy of an aerial robot has to do with the capacity of self-location and perception of the operational environment. In this case, for most applications, GPS (Global Positioning System) still represents the main alternative for addressing the localization problem. Nevertheless, the use of GPS presents some drawbacks, for instance, the precision error can be substantial, and it provides poor operability due to multipath propagation. However, several mission profiles require the UAVs to fly in GPS-challenging or GPS-denied environments, as in natural and urban canyons [3]. The use of range sensors like laser, sonar or radar (see [4,5,6]) allows obtaining knowledge about the environment of the robot. However, this kind of sensor can be expensive and sometimes heavy, and its use in outdoor environments can be somewhat limited. For instance, sonar systems have a limited range of operation. Active laser systems (e.g., LiDAR) represent a very interesting sensing technology; they can operate under any visibility condition (i.e., both day and night, unlike cameras) and can directly provide 3D measurements about the surrounding environment. On the other hand, LiDAR is generally expensive; it can overload the system for certain applications; and it has moving parts, which can generate error.

### 1.1. Related Work

Visual SLAM is a technique that makes use of visual features as landmarks. Visual SLAM is intended to address the navigation problem of a robot moving in a previously unknown environment, while it provides information about the environment, using mainly angular measurements obtained from cameras. Currently, there are two main approaches for implementing vision-based SLAM systems: (i) filtering-based methods [7,8,9] and (ii) the optimization-based methods [10,11]. While the latter approach is shown to give accurate results when the availability of computational power is enough, filtering-based SLAM methods might be still beneficial if limited processing power is available [12].

Some examples of visual SLAM approaches applied to unmanned aerial vehicles are [13,14]. In [15], a visual SLAM proposal that adds inertial measurements given by an Inertial Measurement Unit (IMU) is presented. The potential problem with this kind of approach is related to the fact that the acceleration obtained from the IMU has a dynamic bias, which is difficult to estimate. In [16], an EKF-based (Extended Kalman Filter) method is proposed in order to perform visual odometry with an unmanned aircraft. This method uses inertial sensors, a monocular downward facing camera and a range sensor (sonar altimeter). Unlike vision-based SLAM, in visual odometry approaches, there is not a mapping process. Furthermore, in those approaches, the operating altitude of the UAV is limited by the operating range of the sonar. More recently, new approaches appeared addressing the problem of visual-based navigation in GPS-denied environments, such as [17,18,19].

Multi-robot systems have also received great attention from the robotics research community. This attention is motivated by the inherent versatility that this kind of system has for performing tasks that could be difficult for a single robot. The use of several robots shows advantages like cost reductions, more robustness, better performance and efficiency [20,21]. In the case of the SLAM problem, in [22,23], a centralized architecture is used where all vehicles send their sensor data to a unique Kalman filter. In [16,24,25], the idea of combining monocular SLAM with cooperative, multi-UAV information to improve navigation capabilities in GPS-challenging environments is presented.

In works like [26,27,28,29], it has been shown that 6DOF-SLAM (six degrees of freedom), based only on angular measurements (i.e., monocular SLAM), is a partially observable system that can be applied to both the single-robot case and the multi-robot case. In [30], cooperative localization with visual information is addressed. According to the analysis presented in that work, the proposed system is completely observable. However, in this case, only distances and the relative orientations between robots are estimated. This fact can represent a clear drawback for applications where global measurements of the system are required (e.g., absolute position).

### 1.2. Objectives and Contributions

In this work, nonlinear observability properties of an aerial multi-robot system are analyzed. Based on this analysis, it is shown that the observability properties of this kind of system are improved by the inclusion of measurements of the relative distance between the aerial robots. Furthermore, based on the observability analysis, it is shown that the cooperative approach has theoretical advantages with respect to other configurations like the single-robot monocular SLAM approach. In addition, it is demonstrated that in a system composed of several UAVs, the observation of common landmarks is a sufficient condition in order to propagate through the whole system the information provided by the measurement of the relative distance between two robots. This property allows flexibility in the system as opposed to the absolute need for multiple contacts between robots.

In order to take advantage of all the above theoretical results, in this work, a novel cooperative monocular-based SLAM approach for multi-UAV systems is proposed. The system model is composed of the dynamics of each aerial robot and the Euclidean position of each landmark. The measurements of the system are the projections of the landmarks in the images, provided by the monocular cameras carried individually in every aerial robot. Additionally, as was mentioned before, the availability of some measurements about the relative distance between two robots is assumed.

**Figure 1:**
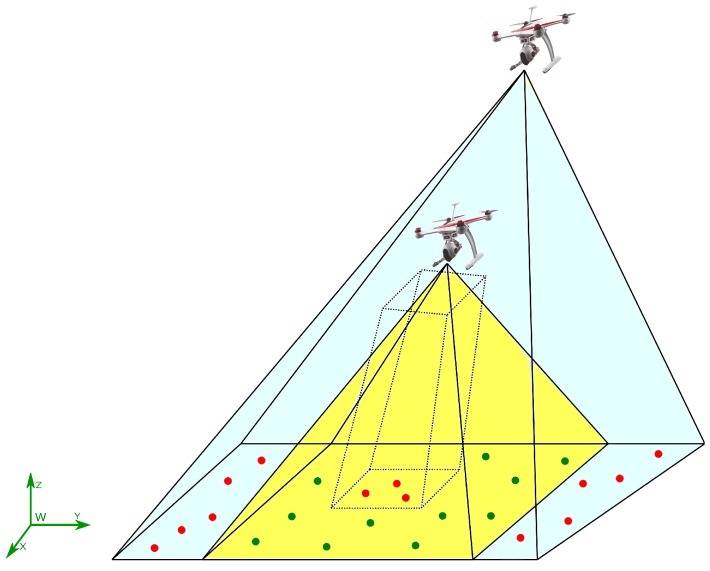
Cooperative monocular-based SLAM.

In order to accomplish the requirement of having measurements of the relative distance between two robots, a technique based on a homography is also presented in this research. The main idea is to exploit the physical structure of the aerial robots in order to obtain measurements of relative distances by means of visual information. In this case, the method is developed assuming a team of quadrotors. It is important to remark that this proposed approach could be also applied to many other aerial platforms. The only requirement for the presented approach is that at least one robot has to be maintained inside the field of view of another aerial robot, while sharing the observation of one common visual landmark (see Figure 1).

The geometric structure of a typical quadrotor is cross-shaped, and therefore, each rotor is mounted at the different ends of the cross. This kind of physical geometry can allow a standard computer vision algorithm to extract and track the centroids of the rotors. In this case, those centroids can be assumed to be coplanar. In order to compute the relative distance from one quadcopter in the field of view of another one, a homography is applied from the camera coordinate reference system of the observing robot to the plane formed by the four rotors of the robot being observed. The information obtained by the homography is fused with the orientation of the observing robot, provided by an IMU, which finally allows one to obtain measurements of relative distances. It is important to note that, based on the theoretical results presented in this work, it should be straightforward to replace the homographic technique used for estimating the relative distance between UAVs by another technique that would provide a similar measurement.

In addition to the benefit of improving the observability of the system, the relative distance obtained between any pair of robots provides metric information of the system, which is an important issue to be addressed in monocular-based systems. For example, in other configurations, the metric information is obtained purely from inertial systems (i.e., monocular/Inertial Navigation Systems (INS) solutions), but inertial sensors present some drawbacks due to the large drift bias, which is inherent to this kind of sensor [31,32].

In the proposed system, in order to take advantage of the multi-UAV cooperative configuration, the initialization process of new map features is carried out through a pseudo-stereo system composed of two monocular cameras mounted on two UAVs respectively (one camera per UAV) that observe common landmarks. This approach allows initializing landmarks with less uncertainty than a pure monocular system since 3D information of the position of landmarks is gathered from the beginning of the observation. It is well known that, in visual SLAM, the initialization process can play an important role in the convergence of the filter. Having a flexible baseline in the pseudo-stereo system allows one to initialize landmarks at distances far away with less uncertainty, unlike stereo systems with a rigid baseline [32] or delayed monocular initialization methods. The above fact allows the proposed cooperative system to have better performance in environments where landmarks are far from the measurement system, contrary to SLAM approaches based on depth cameras, stereo systems, monocular cameras or sonars.

### 1.3. Paper Outline

The document is organized in the following manner: Section 2 presents the specifications of the system; Section 3 presents the nonlinear observability analysis that represents the theoretical basis of the proposed method; Section 4 presents the proposed cooperative approach for monocular-based SLAM; in Section 5, the results obtained from numerical simulations are presented in order to validate the proposal, and finally, in Section 6, some final remarks are given.

## 2. System Specification

In this section, the models used in this work are introduced. The model used for representing the dynamics of a camera carried by a quadcopter is presented. The representation of the landmarks as map features is also defined. The camera projection model used in this work is described. The technique based on homographies that is used for estimating the relative distance between two quadcopters is introduced, as well.

### 2.1. Dynamics of the System

**Figure 2:**
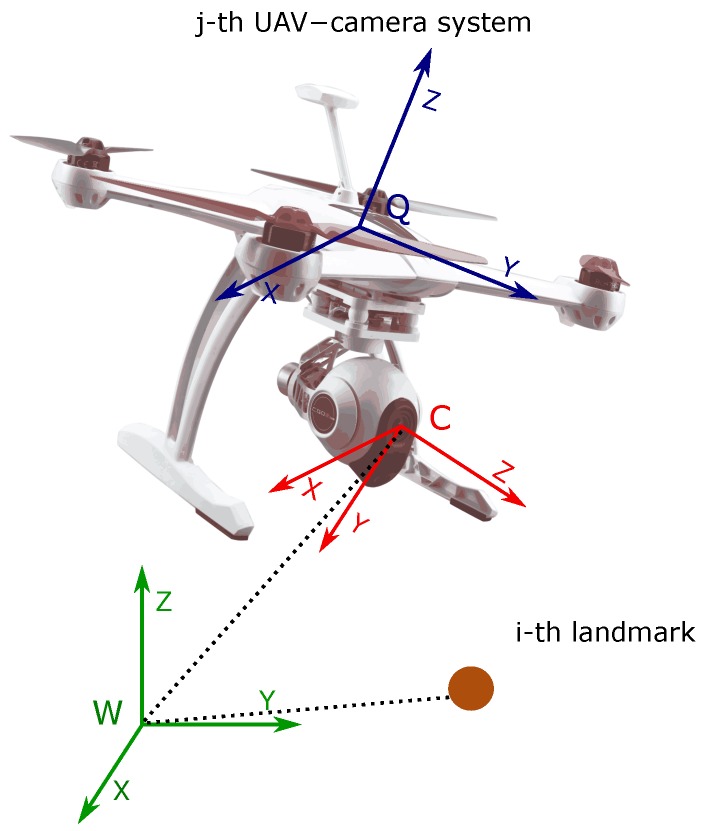
Coordinate reference systems.

Let us consider the following continuous-time model describing the dynamics of the *j*-th UAV-camera system (see Figure 2):

(1) $$\dot{x}=\left[\begin{array}{c} {\dot{x}}_{c}^{j}\\ {\dot{q}}_{c}^{j}\\ {\dot{v}}_{c}^{j}\\ {{\dot{\omega }}_{c}}^{j}\\ {\dot{x}}_{a}^{i} \end{array}\right]=\left[\begin{array}{c} {v_{c}}^{j}\\ \frac{1}{2}{\Omega_{c}}^{j}{q_{c}}^{j}\\ 0_{3\times 1}\\ 0_{3\times 1}\\ 0_{3\times 1} \end{array}\right]$$

where the state vector x is defined by:

(2) $$x={\left[\begin{array}{ccccc} {x_{c}}^{j} & {q_{c}}^{j} & {v_{c}}^{j} & {\omega_{c}}^{j} & {x_{a}}^{i} \end{array}\right]}^{T}$$

With $i=1,\ldots ,n_{1}$, let $n_{1}$ be the number of landmarks, and with $j=1,\ldots ,n_{2}$, let $n_{2}$ be the number of UAV-camera systems.

Additionally, let ${x_{c}}^{j}={\left[\begin{array}{ccc} x_{c}^{j} & y_{c}^{j} & z_{c}^{j} \end{array}\right]}^{T}$ represent the position of the reference system *C* of the *j*-th camera, with respect to the reference system *W*. Let ${q_{c}}^{j}={\left[\begin{array}{cccc} q_{0}^{j} & q_{x}^{j} & q_{y}^{j} & q_{z}^{j} \end{array}\right]}^{T}$ be a unit quaternion representing the orientation of the reference system *C* of the *j*-th camera, with respect to the reference system *W*. Let ${v_{c}}^{j}={\left[\begin{array}{ccc} {\dot{x}}_{c}^{j} & {\dot{y}}_{c}^{j} & {\dot{z}}_{c}^{j} \end{array}\right]}^{T}$ represent the linear velocity of the *j*-th camera. Let ${\omega_{c}}^{j}={\left[\begin{array}{ccc} \omega_{x}^{j} & \omega_{y}^{j} & \omega_{z}^{j} \end{array}\right]}^{T}$ represent the angular velocity of the *j*-th camera. Finally, let ${x_{a}}^{i}={\left[\begin{array}{ccc} x_{a}^{i} & y_{a}^{i} & z_{a}^{i} \end{array}\right]}^{T}$ be the position of the *i*-th landmark with respect to the reference system *W*, defined by its Euclidean parameterization. Furthermore the next definitions should be considered:

(3) $${\Omega_{c}}^{j}=\left[\begin{array}{cc} 0 & \;-{\omega_{c}}^{j^{T}}\\ {\omega_{c}}^{j} & {\left[{\omega_{c}}^{j}\right]}_{\times } \end{array}\right],\;\;\;\;{\left[{\omega_{c}}^{j}\right]}_{\times }=\left[\begin{array}{ccc} 0 & -\omega_{z}^{j} & \omega_{y}^{j}\\ \omega_{z}^{j} & 0 & -\omega_{x}^{j}\\ -\omega_{y}^{j} & \omega_{x}^{j} & 0 \end{array}\right]$$

In (1), it is assumed that every UAV-camera is moving freely in the space with six degrees of freedom: three for translation and three for rotation. Furthermore, note that a non-acceleration model is assumed for UAV-camera systems, and the landmarks are assumed to remain static.

### 2.2. Camera Measurement Model

**Figure 3:**
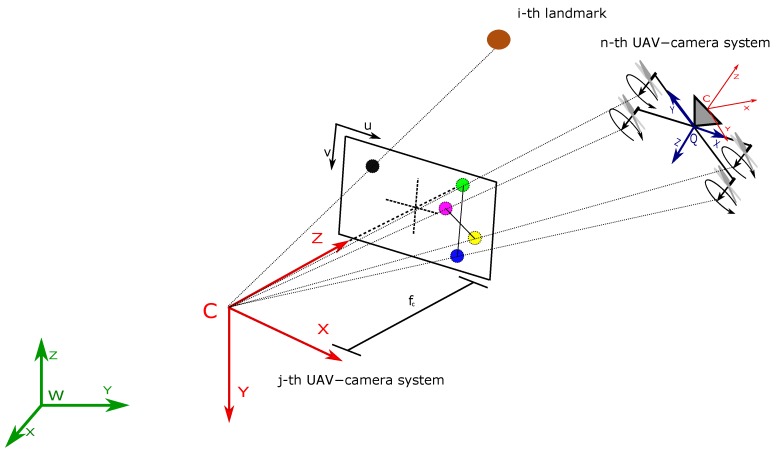
Pinhole camera projection model.

Consider the projection of a specific landmark over the image plane of a camera. Using the pinhole model [33] (see Figure 3), the following expression can be defined:

(4) $$\begin{array}{c} {}^{i}{z_{c}}^{j}={\;}^{i}{h_{c}}^{j}=\left[\begin{array}{c} {}^{i}u_{c}^{j}\\ {}^{i}v_{c}^{j} \end{array}\right]=\frac{1}{{}^{i}z_{d}^{j}}\left[\begin{array}{cc} \frac{f_{c}^{j}}{d_{u}^{j}} & 0\\ 0 & \frac{f_{c}^{j}}{d_{v}^{j}} \end{array}\right]\left[\begin{array}{c} {}^{i}x_{d}^{j}\\ {}^{i}y_{d}^{j} \end{array}\right]+\left[\begin{array}{c} c_{u}^{j}+d_{ur}^{j}+d_{ut}^{j}\\ c_{v}^{j}+d_{vr}^{j}+d_{vt}^{j} \end{array}\right] \end{array}$$

Let ${[}^{i}u_{c}^{j}$,${}^{i}v_{c}^{j}]$ define the coordinates (in pixels) of the projection of the *i*-th landmark over the image of the *j*-th camera. Let $f_{c}^{j}$ be the focal length (in meters) of the *j*-th camera. Let $\left[d_{u}^{j},d_{v}^{j}\right]$ be the conversion parameters (in m/pixel) for the *j*-th camera. Let $\left[c_{u}^{j},c_{v}^{j}\right]$ be the coordinates (in pixels) of the image central point of the *j*-th camera. Let $\left[d_{ur}^{j},d_{vr}^{j}\right]$ be components (in pixels) accounting for the radial distortion of the *j*-th camera. Let $\left[d_{ut}^{j},d_{vt}^{j}\right]$ be components (in pixels) accounting for the tangential distortion of the *j*-th camera. All the intrinsic parameters of the *j*-th camera are assumed to be known by means of some calibration method. Let ${}^{i}{p_{d}}^{j}={\left[\begin{array}{ccc} {}^{i}x_{d}^{j} & {}^{i}y_{d}^{j} & {}^{i}z_{d}^{j} \end{array}\right]}^{T}$ represent the position (in meters) of the *i*-th landmark with respect to the coordinate reference system *C* of the *j*-th camera. Additionally,

(5) $${}^{i}{p_{d}}^{j}={}^{W}{R_{c}}^{j}\left({x_{a}}^{i}-{x_{c}}^{j}\right)$$

Let ${}^{W}{R_{c}}^{j}\left({q_{c}}^{j}\right)\in SO3$ be the rotation matrix, obtained from the quaternion ${q_{c}}^{j}$, that transforms the world coordinate reference system *W* to the coordinate reference system *C* of the *j*-th camera.

### 2.3. Relative Distance Measurement Model

To estimate the relative distance between UAV-camera systems, the physical structure of the aerial robots (quadcopters in this research) is exploited. In this case, the rotors of a quadcopter are considered as highlighted points in the images captured by another quadcopter (see Figure 1 and Figure 3). These points can be considered coplanar in the reference system *Q* of the *n*-th quadcopter. Therefore, knowing their geometry, it is possible to formulate a homography [33]. In order to determine the exact correspondences between the motors’ positions in the image plane and their real positions in reference *Q*, it is assumed that one rotor has a distinguishable color or geometry from the other ones. The other three correspondences can be determined given only the first one because it is also assumed that the quadrotor will not fly upside down. The homographic process will allow estimating the distance between the reference system of the camera to the plane to which the four points belong.

According to Equation (4), the following expression is obtained:

(6) $${}^{j}\gamma_{m}^{n}\left[\begin{array}{c} {}^{j}u_{m}^{n}\\ {}^{j}v_{m}^{n}\\ \;\;1 \end{array}\right]=\left[\begin{array}{cc} {T_{c}}^{j} & 0_{3\times 1} \end{array}\right]{\;}^{j}{E_{c}}^{n}\left[\begin{array}{c} x_{m}^{n}\\ y_{m}^{n}\\ z_{m}^{n}\\ \;\;1 \end{array}\right]$$

With m={1,…,4}, let ${[}^{j}u_{m}^{n}$,${}^{j}v_{m}^{n}]$ define the coordinates (in pixels) of the projection of the *m*-th point of the *n*-th quadcopter over the image of the *j*-th camera. Let $\left[x_{m}^{n},y_{m}^{n},z_{m}^{n}\right]$ represent the position of the *m*-th point with respect to the reference system *Q* of the *n*-th quadcopter, and let ${}^{j}\gamma_{m}^{n}$ be a scale factor. Additionally, it is defined:

(7) $${T_{c}}^{j}=\left[\begin{array}{ccc} \frac{f_{c}^{j}}{d_{u}^{j}} & 0 & c_{u}^{j}+d_{ur}^{j}+d_{ut}^{j}\\ 0 & \frac{f_{c}^{j}}{d_{v}^{j}} & c_{v}^{j}+d_{vr}^{j}+d_{vt}^{j}\\ 0 & 0 & 1 \end{array}\right]$$

(8) $${}^{j}{E_{c}}^{n}=\left[\begin{array}{cc} {}^{j}{R_{q}}^{n} & {}^{j}{d_{q}}^{n}\\ 0_{1\times 3} & 1 \end{array}\right]$$

Let ${}^{j}{d_{q}}^{n}$ be the translation vector from the reference system *Q* of the *n*-th quadcopter to the reference system *C* of the *j*-th camera. Let ${}^{j}{R_{q}}^{n}\in SO3$ be the rotation matrix that transforms the coordinate reference system *Q* of the *n*-th quadcopter to the coordinate reference system *C* of the *j*-th camera. The assumption that the four *m* points are coplanar implies that $z_{m}^{n}=0$; therefore, Equation (6) can take the following form:

(9) $$\left[\begin{array}{c} {}^{j}u_{m}^{n}\\ {}^{j}v_{m}^{n}\\ \;\;1 \end{array}\right]=\frac{1}{{}^{j}\gamma_{m}^{n}}\left[\begin{array}{cc} {T_{c}}^{j} & 0_{3\times 1} \end{array}\right]{\;}^{j}{E_{c}}^{n}\left[\begin{array}{c} x_{m}^{n}\\ y_{m}^{n}\\ \;\;0\\ \;\;1 \end{array}\right]=\left[\begin{array}{ccc} {}^{j}s_{11}^{n} & {}^{j}s_{12}^{n} & {}^{j}s_{13}^{n}\\ {}^{j}s_{21}^{n} & {}^{j}s_{22}^{n} & {}^{j}s_{23}^{n}\\ {}^{j}s_{31}^{n} & {}^{j}s_{32}^{n} & {}^{j}s_{33}^{n} \end{array}\right]\left[\begin{array}{c} x_{m}^{n}\\ y_{m}^{n}\\ \;\;1 \end{array}\right]={\;}^{j}{S_{c}}^{n}\left[\begin{array}{c} x_{m}^{n}\\ y_{m}^{n}\\ \;\;1 \end{array}\right]$$

where ${}^{j}{S_{c}}^{n}$ is a non-singular homogeneous matrix. In this case, it is allowed to scale the matrix in such a way that ${}^{j}s_{33}^{n}=1$. This fact does not affect the projective transformation [33]. Therefore, the matrix ${}^{j}{S_{c}}^{n}$ can be redefined as:

(10) $${}^{j}{S_{c}}^{n}=\left[\begin{array}{ccc} {}^{j}s_{11}^{n} & {}^{j}s_{12}^{n} & {}^{j}s_{13}^{n}\\ {}^{j}s_{21}^{n} & {}^{j}s_{22}^{n} & {}^{j}s_{23}^{n}\\ {}^{j}s_{31}^{n} & {}^{j}s_{32}^{n} & 1 \end{array}\right]=\left[\begin{array}{ccc} {}^{j}{s_{1}}^{n} & {}^{j}{s_{2}}^{n} & {}^{j}{s_{3}}^{n} \end{array}\right]$$

In (10), the values of ${}^{j}{S_{c}}^{n}$ are unknown; therefore, the following equation system can be formed from (9):

(11) $${}^{j}G_{m}^{n}{\;}^{j}{t_{c}}^{n}={\;}^{j}g_{m}^{n}$$

where:

(12) $${}^{j}G_{m}^{n}=\left[\begin{array}{cccccccc} x_{m}^{n} & y_{m}^{n} & 1 & 0 & 0 & 0 & -x_{m}^{n}{\;}^{j}u_{m}^{n} & -y_{m}^{n}{\;}^{j}u_{m}^{n}\\ 0 & 0 & 0 & x_{m}^{n} & y_{m}^{n} & 1 & -x_{m}^{n}{\;}^{j}v_{m}^{n} & -y_{m}^{n}{\;}^{j}v_{m}^{n} \end{array}\right]$$

(13) $${}^{j}{t_{c}}^{n}={\left[\begin{array}{cccccccc} {}^{j}s_{11}^{n} & {}^{j}s_{12}^{n} & {}^{j}s_{13}^{n} & {}^{j}s_{21}^{n} & {}^{j}s_{22}^{n} & {}^{j}s_{23}^{n} & {}^{j}s_{31}^{n} & {}^{j}s_{32}^{n} \end{array}\right]}^{T}$$

(14) $${}^{j}g_{m}^{n}={\left[\begin{array}{cc} {}^{j}u_{m}^{n} & {}^{j}v_{m}^{n} \end{array}\right]}^{T}$$

Considering the projection of the four points, the solution to the system can be given as follows:

(15) $${}^{j}{t_{c}}^{n}={{(}^{j}{G_{c}}^{n})}^{-1}{\;}^{j}{g_{c}}^{n}$$

with:

(16) $$\begin{array}{c} {}^{j}{G_{c}}^{n}={\left[\begin{array}{cccc} {}^{j}G_{1}^{n} & {}^{j}G_{2}^{n} & {}^{j}G_{3}^{n} & {}^{j}G_{4}^{n} \end{array}\right]}^{T}{\;}^{j}{g_{c}}^{n}={\left[\begin{array}{cccc} {}^{j}g_{1}^{n} & {}^{j}g_{2}^{n} & {}^{j}g_{3}^{n} & {}^{j}g_{4}^{n} \end{array}\right]}^{T} \end{array}$$

From the method proposed in [34], where the orthonormality property of a rotation matrix is exploited and knowing the intrinsic parameters of the camera, ${}^{j}{R_{q}}^{n}$ and ${}^{j}{d_{q}}^{n}$ can be computed from (15) as follows:

(17) $${}^{j}{R_{q}}^{n}=\left[\begin{array}{ccc} {}^{j}r_{1}^{n} & {}^{j}r_{2}^{n} & {}^{j}r_{1}^{n}\times {\;}^{j}r_{2}^{n} \end{array}\right]$$

(18) $${}^{j}{d_{q}}^{n}={\;}^{j}r_{3}^{n}$$

with:

(19) $$\left[\begin{array}{ccc} {}^{j}r_{1}^{n} & {}^{j}r_{2}^{n} & {}^{j}r_{3}^{n} \end{array}\right]=\frac{1}{{}^{j}\delta_{c}^{n}}{\left({T_{c}}^{j}\right)}^{-1}{\;}^{j}{S_{c}}^{n}$$

and:

(20) $${}^{j}\delta_{c}^{n}=\left|\left|{\left({T_{c}}^{j}\right)}^{-1}{\;}^{j}s_{1}^{n}\right|\right|=\left|\left|{\left({T_{c}}^{j}\right)}^{-1}{\;}^{j}s_{2}^{n}\right|\right|$$

Finally, the distance between the *j*-th camera and the *n*-th camera can be computed from:

(21) $${}^{j}{z_{r}}^{n}{=}^{j}{h_{r}}^{n}={x_{c}}^{n}-{x_{c}}^{j}=\;{{(}^{W}{R_{c}}^{j})}^{T}{(}^{j}{R_{q}}^{j}\;{d_{c}}^{n}+{\;}^{j}{d_{q}}^{n})$$

where ${d_{c}}^{n}$ is the translation vector of the reference system *Q* to the reference system *C* of the *n*-th UAV-camera system. This parameter is obtained by a camera-robot calibration process. The rotation matrix ${}^{W}{R_{c}}^{i}$ can be obtained from an Attitude and Heading Reference System (AHRS) or an inertial measurement unit [35,36] mounted on board the *j*-th UAV-camera system.

## 3. Observability Analysis

In this section, the nonlinear observability properties of an aerial multi-robot system are studied. Observability is an inherent property of a dynamic system and has an important role in the accuracy of its estimation process; moreover, this fact has important consequences in the context of SLAM.

A system is defined as observable if the initial state $x_{0}$, at any initial time $t_{0}$, can be determined given the state transition and observation models of the system and observations $z[t_{0},t]$, from time $t_{0}$ to a finite time *t*. In [37], it is demonstrated that a non-linear system is locally weakly observable if the observability rank condition rank(O)=dim(x) is verified, where O is the observability matrix.

As previously mentioned, 6DOF-monocular SLAM represents a kind of partially-observable system with a high number of unobservable modes and states that can be applied to both the single-robot case and the multi-robot case. The following references are examples of works where the problem of the observability of 6DOF-monocular SLAM systems has also been studied, such as [26,27,28,29].

For the analysis developed in this work, and for the sake of simplicity, the system (1) is redefined as:

(22) $$\dot{x}=f\left(x,t\right)=\left[\begin{array}{c} {\dot{x}}_{c}^{j}\\ {\dot{\lambda }}_{c}^{j}\\ {\dot{v}}_{c}^{j}\\ {{\dot{\omega }}_{c}}^{j}\\ {\dot{x}}_{a}^{i} \end{array}\right]=\left[\begin{array}{c} {v_{c}}^{j}\\ {\omega_{c}}^{j}\\ 0_{3\times 1}\\ 0_{3\times 1}\\ 0_{3\times 1} \end{array}\right]$$

Let ${\lambda_{c}}^{j}={\left[\begin{array}{ccc} \phi_{c}^{j} & \theta_{c}^{j} & \psi_{c}^{j} \end{array}\right]}^{T}$ be the Euler angles of the *j*-th camera with respect to the coordinate system *W*.

The observability matrix O can be computed as:

(23) $$O={\left[\begin{array}{cccc} \frac{\partial (L_{f}^{0}{(}^{i}{h_{c}}^{j}))}{\partial x} & \frac{\partial (L_{f}^{1}{(}^{i}{h_{c}}^{j}))}{\partial x} & \frac{\partial (L_{f}^{0}{(}^{j}{h_{r}}^{n}))}{\partial x} & \frac{\partial (L_{f}^{1}{(}^{j}{h_{r}}^{n}))}{\partial x} \end{array}\right]}^{T}$$

Let $L_{f}^{s}h$ be the *s*-th-order Lie derivative [38] of the scalar field h with respect to the vector field f. For example, in (23), the zero-order and first-order Lie derivatives are used for each measurement.

For the measurement given by a monocular camera, according to (4) and (22), the following zero-order Lie derivative can be defined:

(24) $$\frac{\partial (L_{f}^{0}{(}^{i}{h_{c}}^{j}))}{\partial x}=\left[\begin{array}{ccccccc} 0_{2\times 12(j-1)} & {H_{x}}^{j} & 0_{2\times 12(n_{2}-j)} & | & 0_{2\times 3(i-1)} & {H_{a}}^{i} & 0_{2\times 3(n_{1}-i)} \end{array}\right]$$

where:

(25) $${H_{x}}^{j}=H_{c}\left[\begin{array}{ccc} {-}^{W}{R_{c}}^{j} & \lfloor^{i}{p_{d}}^{j}\;\times \rfloor & 0_{2\times 6} \end{array}\right]$$

(26) $${H_{a}}^{i}={H_{c}}^{w}{R_{c}}^{w}$$

and:

(27) $$H_{c}=\frac{f_{c}^{j}}{{}^{i}z_{d}^{j^{2}}}\left[\begin{array}{ccc} {}^{i}z_{d}^{j} & 0 & {-}^{i}x_{d}^{j}\\ 0 & {}^{i}z_{d}^{j} & {-}^{i}y_{d}^{j} \end{array}\right]$$

Note that ⌊·×⌋ denotes the antisymmetric matrix of the vector (·). The first-order Lie derivative can also be defined in the following:

(28) $$\frac{\partial (L_{f}^{1}{(}^{i}{h_{c}}^{j}))}{\partial x}=\left[\begin{array}{ccccccc} 0_{2\times 12(j-1)} & {H_{\mathrm{dx}}}^{j} & 0_{2\times 12(n_{2}-j)} & | & 0_{2\times 3(i-1)} & {H_{\mathrm{da}}}^{i} & 0_{2\times 3(n_{1}-i)} \end{array}\right]$$

where:

(29) $${H_{\mathrm{dx}}}^{j}=\left[\begin{array}{cccc} X^{j} & \Psi^{j} & -{H_{c}}^{W}{R_{c}}^{j} & H_{c}\lfloor^{i}{p_{d}}^{j}\;\times \rfloor \end{array}\right]$$

(30) $$\begin{array}{c} {H_{\mathrm{da}}}^{i}={\left[\begin{array}{ccc} H_{c}^{1} & H_{c}^{2} & H_{c}^{3} \end{array}\right]}^{W}{R_{c}}^{j}\left({-}^{W}{R_{c}}^{j}\;{v_{c}}^{j}+\lfloor^{i}{p_{d}}^{j}\;\times \rfloor {\omega_{c}}^{j}\right)-H_{c}{\left\lfloor {\omega_{c}}^{j}\;\times \right\rfloor }^{W}{R_{c}}^{j} \end{array}$$

with:

(31) $$X^{j}=-{H_{\mathrm{da}}}^{i}$$

(32) $$\begin{array}{c} \Psi^{j}=\left[\begin{array}{ccc} H_{c}^{1} & H_{c}^{2} & H_{c}^{3} \end{array}\right]\lfloor^{i}{p_{d}}^{j}\;\times \rfloor \left({-}^{W}{R_{c}}^{j}\;v_{c}^{j}+\lfloor^{i}{p_{d}}^{j}\;\times \rfloor {\omega_{c}}^{j}\right)-H_{c}\lfloor^{W}{R_{c}}^{j}\;{v_{c}}^{j}\;\times \rfloor -H_{c}\left\lfloor {\omega_{c}}^{j}\;\times \right\rfloor \lfloor^{i}{p_{d}}^{j}\;\times \rfloor \end{array}$$

and:

(33) $$H_{c}^{1}=\frac{f_{c}^{j}}{{}^{i}z_{d}^{j^{2}}}\left[\begin{array}{ccc} 0 & 0 & -1\\ 0 & 0 & 0 \end{array}\right]$$

(34) $$H_{c}^{2}=\frac{f_{c}^{j}}{{}^{i}z_{d}^{j^{2}}}\left[\begin{array}{ccc} 0 & 0 & 0\\ 0 & 0 & -1 \end{array}\right]$$

(35) $$H_{c}^{3}=\frac{f_{c}^{j}}{{}^{i}z_{d}^{j^{3}}}\left[\begin{array}{ccc} {-}^{i}z_{d}^{j} & 0 & 2^{i}x_{d}^{j}\\ 0 & {-}^{i}z_{d}^{j} & 2^{i}y_{d}^{j} \end{array}\right]$$

Considering the case where relative measurements of the distance between robots are available, the following statement can be defined from (21) and (22):

For the zero-order Lie derivative, if j<n (the index of the observing robot is lesser than the index of the observed robot):

(36) $$\frac{\partial (L_{f}^{0}{(}^{j}{h_{r}}^{n}))}{\partial x}=\left[\begin{array}{ccccccc} 0_{3\times 12(j-1)} & -{M_{x}}^{j} & 0_{3\times 12(n-j-1)} & {M_{x}}^{n} & 0_{3\times 12(n_{2}-n)} & | & 0_{3n_{1}} \end{array}\right]$$

On the other hand, if j>n, then:

(37) $$\frac{\partial (L_{f}^{0}{(}^{j}{h_{r}}^{n}))}{\partial x}=\left[\begin{array}{ccccccc} 0_{3\times 12(n-1)} & {M_{x}}^{n} & 0_{3\times 12(j-n-1)} & -{M_{x}}^{j} & 0_{3\times 12(n_{2}-j)} & | & 0_{3n_{1}} \end{array}\right]$$

and:

(38) $${M_{x}}^{j,n}=\left[\begin{array}{cc} I_{3} & 0_{3\times 9} \end{array}\right]$$

where I is the identity matrix.

For the first-order Lie derivative, if j<n:

(39) $$\frac{\partial (L_{f}^{1}{(}^{j}{h_{r}}^{n}))}{\partial x}=\left[\begin{array}{ccccccc} 0_{3\times 12(j-1)} & -{M_{\mathrm{dx}}}^{j} & 0_{3\times 12(n-j-1)} & {M_{\mathrm{dx}}}^{n} & 0_{3\times 12(n_{2}-n)} & | & 0_{3n_{1}} \end{array}\right]$$

On the other hand, if j>n (the index of the observing robot is higher than the index of the observed robot), then:

(40) $$\frac{\partial (L_{f}^{1}{(}^{j}{h_{r}}^{n}))}{\partial x}=\left[\begin{array}{ccccccc} 0_{3\times 12(n-1)} & {M_{\mathrm{dx}}}^{n} & 0_{3\times 12(j-n-1)} & -{M_{\mathrm{dx}}}^{j} & 0_{3\times 12(n_{2}-j)} & | & 0_{3n_{1}} \end{array}\right]$$

with

(41) $${M_{\mathrm{dx}}}^{j,n}=\left[\begin{array}{ccc} 0_{3\times 6} & I_{3} & 0_{3} \end{array}\right]$$

With the above considerations, the observability matrix for the proposed system (22) can be defined as follows:

(42) $$O=\left[\begin{array}{ccccccc} 0_{2\times 12(j-1)} & {H_{x}}^{j} & 0_{2\times 12(n_{2}-j)} & | & 0_{2\times 3(i-1)} & {H_{a}}^{i} & 0_{2\times 3(n_{1}-i)}\\ 0_{2\times 12(j-1)} & {H_{\mathrm{dx}}}^{j} & 0_{2\times 12(n_{2}-j)} & | & 0_{2\times 3(i-1)} & {H_{\mathrm{da}}}^{i} & 0_{2\times 3(n_{1}-i)}\\ 0_{3\times 12(j-1)} & -{M_{x}}^{j} & 0_{3\times 12(n-j-1)} & {M_{x}}^{n} & 0_{3\times 12(n_{2}-n)} & | & 0_{3n_{1}}\\ 0_{3\times 12(j-1)} & -{M_{\mathrm{dx}}}^{j} & 0_{3\times 12(n-j-1)} & {M_{\mathrm{dx}}}^{n} & 0_{3\times 12(n_{2}-n)} & | & 0_{3n_{1}}\\ & & & \vdots & & & \\ 0_{3\times 12(n-1)} & {M_{x}}^{n} & 0_{3\times 12(j-n-1)} & -{M_{x}}^{j} & 0_{3\times 12(n_{2}-j)} & | & 0_{3n_{1}}\\ 0_{3\times 12(n-1)} & {M_{\mathrm{dx}}}^{n} & 0_{3\times 12(j-n-1)} & -{M_{\mathrm{dx}}}^{j} & 0_{3\times 12(n_{2}-j)} & | & 0_{3n_{1}} \end{array}\right]$$

The maximum rank of the observability matrix (42) is $rank\left(O\right)=3n_{1}+12n_{2}-3$, where $n_{1}$ is the number of landmarks being measured and $n_{2}$ is the number of robots. $n_{1}$ is multiplied by three, since this is the number of states per landmark given by the Euclidean parametrization. $n_{2}$ is multiplied by 12, since this is the number of states per robot given by its global position, orientation (Euler angles) and its derivatives. Therefore, O will be rank deficient (rank(O)<dim(x)).

The unobservable modes are spanned by the right nullspace basis of the observability matrix O; therefore:

(43) $$N=\mathrm{null}\left(O\right)=\left[\begin{array}{c} \;\;I_{3}^{1}\\ 0_{9\times 3}^{1}\\ \;\;\;\vdots \\ \;\;I_{3}^{j}\\ 0_{9\times 3}^{j}\\ -\\ \;\;I_{3}^{1}\\ \;\;\;\vdots \\ \;\;I_{3}^{i} \end{array}\right]$$

It is straightforward to verify that the right nullspace basis of O spans for N (i.e., ON=0).

From (43), it can be seen that the system is partially observable and that the unobservable modes cross with the states that correspond to the global position of the robots and the landmarks; these states are unobservable. An important conclusion is that all the vectors of the right null space basis are orthogonal with the rest of the states, and therefore, these states are completely observable.

The results of the observability analysis are summarized in Table 1.

**Figure 4:**
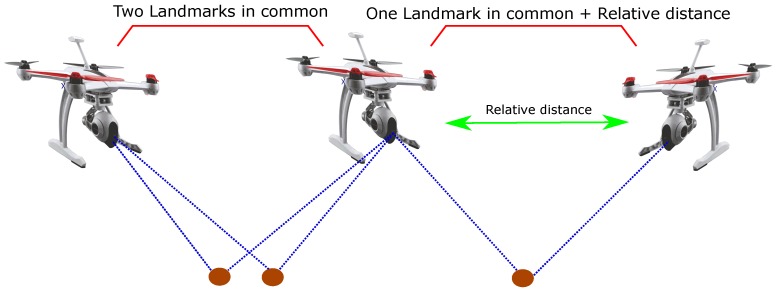
Requirements to obtain the results of the observability analysis for the proposed system.

Some important remarks on the analysis can be extracted:

- In order to obtain the previous results, it is necessary to link the members of the multi-UAV system through the measurements (see Figure 4). In other words, (i) a robot needs to share the observation of at least two landmarks with another robot or (ii) a robot needs to measure its relative distance with respect to another robot in addition to both observing one landmark in common.
- A single measurement of the relative distance between two robots represents a sufficient condition to obtain the previous results (see Figure 4).
- Adding Lie derivatives of higher order to the observability matrix does not improve the results.

From the above results, it can be concluded that the proposed cooperative system, although still partially observable, considerably reduces the unobservable modes and states with respect to the 6DOF-monocular SLAM system. This contribution represents an advantage to improve the accuracy and consistency in the estimation process.

## 4. EKF-Cooperative Monocular SLAM

**Figure 5:**
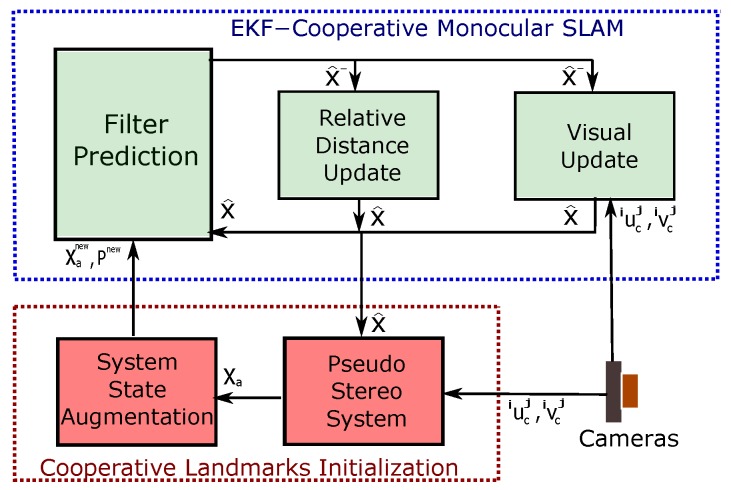
Block diagram showing the architecture of the system: EKF-cooperative monocular SLAM.

In this section, the proposed monocular cooperative SLAM algorithm, based on an Extended Kalman Filter (EKF), is presented. Figure 5 shows the architecture of the proposed system.

### 4.1. EKF-SLAM

According to (1), the discrete system state to be estimated is defined by:

(44) $$x_{k}=f\left(x_{k-1},n_{k-1}\right)=\left[\begin{array}{c} {x_{c}}_{k}^{j}\\ {q_{c}}_{k}^{j}\\ {v_{c}}_{k}^{j}\\ {\omega_{c}}_{k}^{j}\\ {x_{a}}_{k}^{i} \end{array}\right]=\left[\begin{array}{c} \;\;{x_{c}}_{k-1}^{j}+\left({v_{c}}_{k-1}^{j}\right)\Delta t\\ {q_{c}}_{k-1}^{j}\times q\left(\left({\omega_{c}}_{k-1}^{j}\right)\Delta t\right)\\ \;\;\;\;\;\;\;\;\;\;\;\;{v_{c}}_{k-1}^{j}+{\zeta_{c}}_{k-1}^{j}\\ \;\;\;\;\;\;\;\;\;\;\;\;{\omega_{c}}_{k-1}^{j}+{\eta_{c}}_{k-1}^{j}\\ \;\;\;\;\;\;\;\;\;\;\;\;\;\;\;\;{x_{a}}_{k-1}^{i} \end{array}\right]$$

(45) $$n_{k}=\left[\begin{array}{c} {\zeta_{c}}_{k}^{j}\\ {\eta_{c}}_{k}^{j} \end{array}\right]=\left[\begin{array}{c} {a_{c}}^{j}\Delta t\\ {\alpha_{c}}^{j}\Delta t \end{array}\right]$$

with system measurements defined according to (4) and (21), as:

(46) $$z_{k}=h\left(x_{k},r_{k}\right)=\left[\begin{array}{c} {}^{i}{h_{c}}_{k}^{j}{+}^{i}{r_{c}}_{k}^{j}\\ {}^{j}{h_{r}}_{k}^{n}{+}^{j}{r_{e}}_{k}^{n} \end{array}\right]$$

(47) $$r_{k}=\left[\begin{array}{c} {}^{i}{r_{c}}_{k}^{j}\\ {}^{j}{r_{e}}_{k}^{n} \end{array}\right]$$

Let ${a_{c}}^{j}$ and ${\alpha_{c}}^{j}$ represent unknown linear and angular accelerations that are assumed to have a Gaussian distribution with zero mean. Let $n_{k}∼N\left(0,Q_{k}\right)$ and $r_{k}∼N\left(0,R_{k}\right)$ be the noise vectors that affect the state and the measurement, which are assumed to be mutually uncorrelated. Let Δt be the differential of time and *k* the sample step. Note that in this work, for simplicity, a Gaussian random process is used for propagating the velocity of the vehicle. However, a feasible alternative could be to use the dynamical model of the aircraft instead. However, this approach commonly requires having considerable knowledge of the specific physics of each aerial vehicle where the proposed method would have to be applied.

The prediction stage of the EKF is defined by:

(48) $${\hat{x}}_{k}^{-}=f\left({\hat{x}}_{k-1},0\right)$$

(49) $$P_{k}^{-}=A_{k}P_{k-1}A_{k}^{T}+W_{k}Q_{k-1}W_{k}^{T}$$

The correction stage of the EKF is defined by:

(50) $${\hat{x}}_{k}={\hat{x}}_{k}^{-}+K_{k}\left(z_{k}-h\left({\hat{x}}_{k}^{-},0\right)\right)$$

(51) $$P_{k}=\left(I-K_{k}C_{k}\right)P_{k}^{-}$$

with:

(52) $$K_{k}=P_{k}^{-}C_{k}^{T}{\left(C_{k}P_{k}^{-}C_{k}^{T}+V_{k}R_{k}V_{k}^{T}\right)}^{-1}$$

and:

(53) $$\begin{array}{c} A_{k}=\frac{\partial f}{\partial x}\left({\hat{x}}_{k-1},0\right)\;C_{k}=\frac{\partial h}{\partial x}\left({\hat{x}}_{k}^{-},0\right)\\ W_{k}=\frac{\partial f}{\partial n}\left({\hat{x}}_{k-1},0\right)\;V_{k}=\frac{\partial h}{\partial r}\left({\hat{x}}_{k}^{-},0\right) \end{array}$$

P is the covariance matrix of the system state, and K is the Kalman gain.

### 4.2. Initialization of Map Features

Taking advantage of the multi-UAV cooperative system, the initialization process of new map features is carried out through a pseudo-stereo system composed of two different UAV cameras that observe common landmarks. This fact allows initializing the landmarks with less uncertainty since 3D information of the position of the landmarks is gathered from the beginning. The three-dimensional data obtained by the pseudo-stereo system can improve the information obtained by other sensors. For example, the traditional fixed stereo system has a limited operating range due to the fixed baseline between the cameras.

The process of initialization is carried out when a new landmark is observed by two cameras, and if this condition is fulfilled, then the landmark can be initialized by means of a linear triangulation. In this case, the measurement is computed using the *a posteriori* values obtained in the correction stage of the EKF.

According to (4) and (6), the following expression can be defined in homogeneous coordinates:

(54) $${}^{i}\gamma_{c}^{j}\left[\begin{array}{c} {}^{i}u_{c}^{j}\\ {}^{i}v_{c}^{j}\\ \;\;1 \end{array}\right]=\left[\begin{array}{cc} {T_{c}}^{j} & 0_{3\times 1} \end{array}\right]{\hat{E}}_{c}^{j}\left[\begin{array}{c} {x_{a}}^{i}\\ \;\;1 \end{array}\right]$$

where:

(55) $${\hat{E}}_{c}^{j}=\left[\begin{array}{cc} {}^{W}{\hat{R}}_{c}^{j} & {\hat{x}}_{c}^{j}\\ 0_{1\times 3} & 1 \end{array}\right]$$

Using (54) and considering the projection onto two any UAV cameras, a linear system can be formed in order to estimate ${x_{a}}^{i}$:

(56) $$D^{i}{x_{a}}^{i}=b^{i}\;{x_{a}}^{i}=D^{i^{†}}b^{i}$$

where $D^{i^{†}}$ is the Moore–Penrose right pseudo-inverse matrix of $D^{i}$, and:

(57) $$\begin{array}{c} D^{i}=\left[\begin{array}{ccc} k_{31}^{j}{\;}^{i}u_{c}^{j}-k_{11}^{j} & k_{32}^{j}{\;}^{i}u_{c}^{j}-k_{12}^{j} & k_{33}^{j}{\;}^{i}u_{c}^{j}-k_{13}^{j}\\ k_{31}^{j}{\;}^{i}v_{c}^{j}-k_{21}^{j} & k_{32}^{j}{\;}^{i}v_{c}^{j}-k_{22}^{j} & k_{33}^{j}{\;}^{i}v_{c}^{j}-k_{23}^{j} \end{array}\right]\\ b^{i}=\left[\begin{array}{c} k_{14}^{j}-k_{34}^{j}{\;}^{i}u_{c}^{j}\\ k_{24}^{j}-k_{34}^{j}{\;}^{i}v_{c}^{j} \end{array}\right] \end{array}$$

with:

(58) $$\left[\begin{array}{cc} {T_{c}}^{j} & 0_{3\times 1} \end{array}\right]{\hat{E}}_{c}^{j}=\left[\begin{array}{cccc} k_{11}^{j} & k_{12}^{j} & k_{13}^{j} & k_{14}^{j}\\ k_{21}^{j} & k_{22}^{j} & k_{23}^{j} & k_{24}^{j}\\ k_{31}^{j} & k_{32}^{j} & k_{33}^{j} & k_{34}^{j} \end{array}\right]$$

When a new landmark is initialized, the system state x is augmented by: $x={\left[\begin{array}{cccccc} {x_{c}}^{j} & {q_{c}}^{j} & {v_{c}}^{j} & {\omega_{c}}^{j} & {x_{a}}^{i} & {x_{a}}^{new} \end{array}\right]}^{T}$.

The new covariance matrix $P_{new}$ is computed by:

(59) $$P_{new}=\Delta J\left[\begin{array}{cc} P & 0\\ 0 & {}^{i}R^{j} \end{array}\right]=\Delta J^{T}$$

where ΔJ is the Jacobian for the initialization function and ${}^{i}R^{j}$ is the measurement noise covariance matrix for ${(}^{i}u_{c}^{j}{,}^{i}v_{c}^{j})$.

#### Map Management

The real-time feasibility of EKF-based visual SLAM systems has been proven since early works like [39]. Nevertheless, it is well known that due to the nature of the Kalman filter, in SLAM, the system state can always reach a size that will make it impossible to maintain a real-time performance for a given hardware. In this sense, this work is mainly intended to address the local navigation problem, that is the proposed system is intended to be applied in scenarios involving flight trajectories relatively near the origin of the navigation frame. Therefore, old features can be removed from the system state and covariance matrix, to prevent the system state from reaching a size that affects the computational performance.

On the other hand, although large-scale SLAM and loop-closing are not considered in this work, it is important to note that a SLAM framework that works reliably in a local way can be applied to large-scale problems using different methods, such as sub-mapping or graph-based global optimization [12].

## 5. Computer Simulations Results

In this section, computer simulation results are presented. The computer simulations were performed in order to validate the performance of the proposed method. A MATLAB${}^{®}$ implementation was used for this purpose.

With the intention of making an exhaustive analysis of the performance of the proposed system, a comparison is carried out with respect to the other three typical single-robot SLAM configurations. The comparison allows one to note the advantages and drawbacks of multi-UAV systems compared with single robot systems.

For the computer simulations setup, two quadcopters equipped with an onboard monocular camera are simulated, while moving maintaining a stable flight formation. In this case, a Quadcopter (Quad 2) navigates over the other (Quad 1) at an arbitrary relative distance. In the computer simulations, it is considered that Quad 1 remains all the time inside the visual field of Quad 2. It is also assumed that there exist some landmarks observed in common by the cameras of both quadcopters.

The characteristics of the three SLAM configurations used for the comparison are described below:

1. The first configuration to be compared is monocular SLAM. In this case, the estimates are obtained from the monocular camera carried by Quad 1. The Monocular SLAM approach used to implement this configuration is based on the method proposed in [40]. In this method, the map features are parametrized with the inverse depth parametrization. Both the initialization and update process are performed by means of the monocular measurements. The metric scale of the estimates cannot be retrieved when only monocular vision is used. For this reason, for this configuration, it is assumed that the position of the landmarks seen in the first frame (at the beginning of the flight trajectory) is perfectly known.
2. The second configuration to be compared is stereo SLAM. In this case, the estimates are obtained from a stereo system, with a baseline of 15 cm, carried by Quad 1. In this method, the map features are parametrized with the Euclidean parametrization. The feature initialization process is carried out directly by means of the 3D information provided by the stereo system. The state update is also performed using the stereo measurements.
3. The third configuration to be compared is a hybrid system stereo-monocular SLAM. In this case, the estimates are obtained from a stereo system, with a baseline of 15 cm, carried by Quad 1. In this method, the map features are parametrized with the Euclidean parametrization. The features initialization process is carried out directly by means of the 3D information provided by the stereo system. Unlike the second configuration, in this case, the state update is performed through monocular measurements obtained from one of the cameras of the stereo system.

In computer simulations, it is assumed that the initial condition of the quadcopter states is known with certainty. In order to emulate uncertainty, Gaussian noise with $\sigma_{c}=3$ pixels is added to the measurements given by the cameras. The measurements from the cameras are taken with a frequency of 10 Hz. The intrinsic parameters used for the cameras are $f_{c}^{j}/d_{u}^{j}=f_{c}^{j}/d_{v}^{j}=200.1$ and $c_{u}^{j}=c_{v}^{j}=500$. The environment is composed of 3D points, randomly distributed over the ground. Furthermore, it is assumed that the camera can detect and track visual features without error, avoiding the data association problem. Furthermore, the problem of the influence of the estimates on the control system was not considered. In other words, an almost perfect control over the vehicle is assumed. The trajectory followed by the vehicles begins near the ground, then it moves away from the initial position taking a higher altitude as the trajectory progresses.

The average NEES (Normalized Estimation Error Squared [41]) over $n_{3}$ Monte Carlo runs was used in order to evaluate the consistency of each method, as proposed in [42]. The NEES is estimated as follows:

(60) $$\epsilon_{k}={\left[\begin{array}{c} x_{k}-{\hat{x}}_{k} \end{array}\right]}^{T}P_{k}^{-1}\left[\begin{array}{c} x_{k}-{\hat{x}}_{k} \end{array}\right]$$

The average NEES is computed from:

(61) $${\bar{\epsilon }}_{k}=\frac{1}{n_{3}}\sum_{r=1}^{n_{3}}\epsilon_{k}^{r}$$

**Figure 6:**
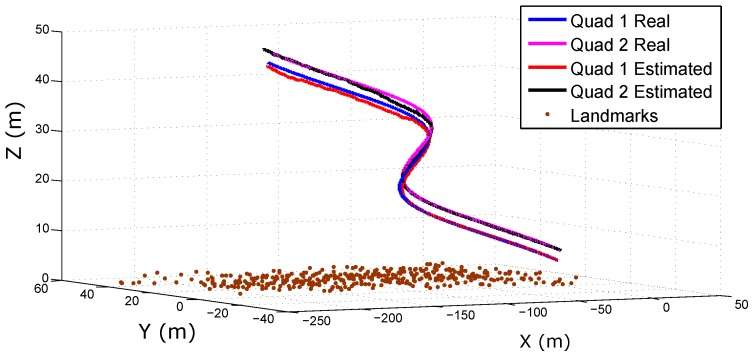
Estimated trajectories of the Quadcopters (Quad) obtained with the cooperative method.

**Figure 7:**
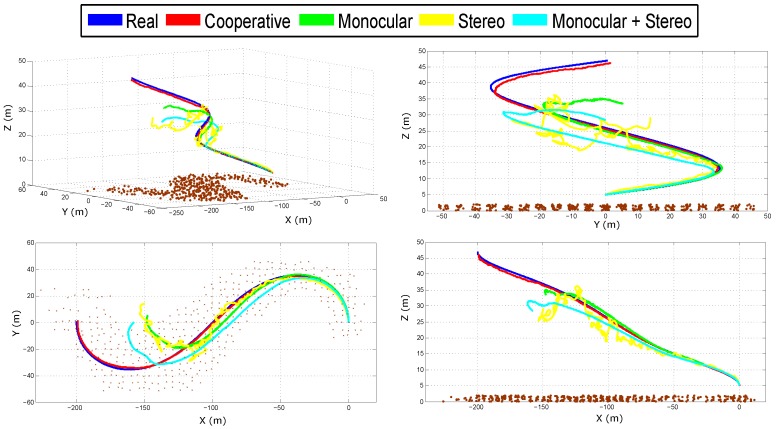
Estimated trajectory of Quad 1 obtained with all the configurations.

Figure 6 shows the real and estimated trajectory obtained from the cooperative system. Figure 7 shows the real and estimated trajectory obtained with all the configurations. Note that in this case, only the trajectory of Quad 1 is presented. In this simulation, it can be seen that as the trajectory evolves, the error considerably increases for the single-robot configurations. On the other hand, for the proposed (cooperative) method, the error is better bounded.

**Figure 8:**
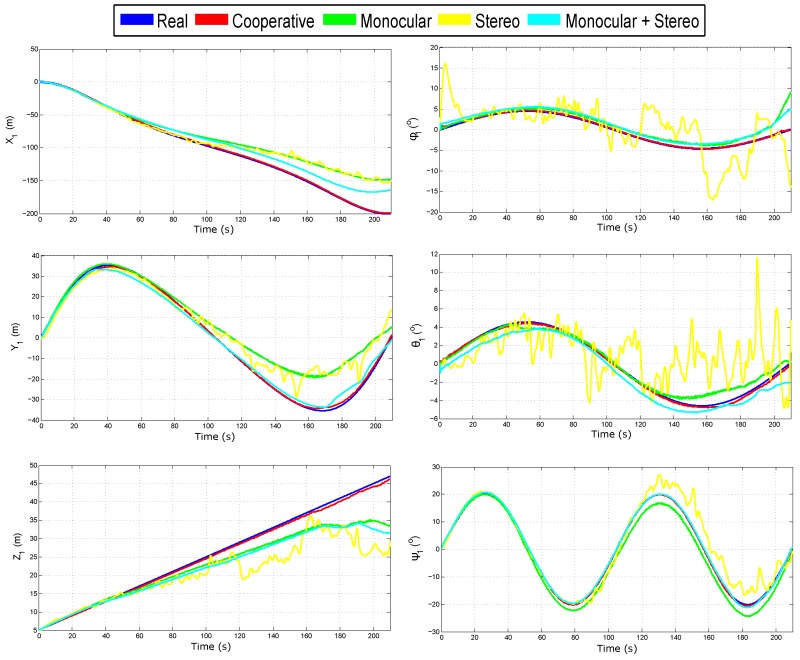
Estimated state of Quad 1.

Figure 8 shows the evolution over time of the real and estimated states (position and orientation) for Quad 1. In this case, the initial results are confirmed. The results of the estimated state of Quad 2 are not shown, but they are closely similar to those presented for Quad 1. Table 2 summarizes the Mean Squared Error (MSE) for the position in the three axes of Quad 1.

**Figure 9:**
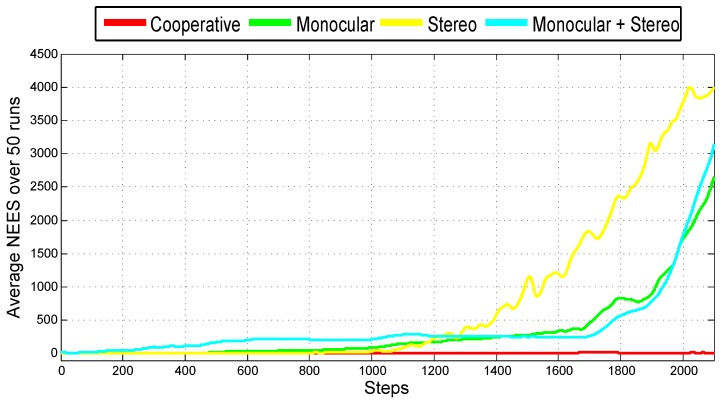
Average Normalized Estimation Error Squared (NEES) obtained with the four configurations.

Figure 9 shows the average NEES over 50 Monte Carlo runs obtained for each method. The average NEES is calculated taking into account the twelve variables that define the complete state of the vehicle (position, orientation, linear velocity and angular velocity). It is very interesting to note how the consistency of the filter considerably degenerates in the three cases of the single-robot configurations. On the other hand, for the cooperative case, the consistency of the filter remains practically stable.

**Figure 10:**
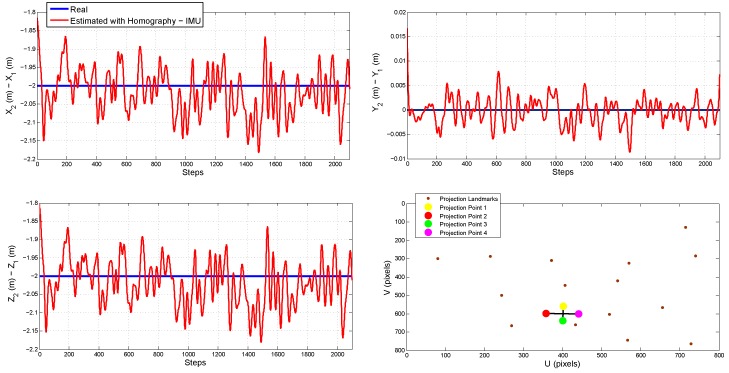
Estimation of the relative distances between the flying vehicles by means of homographies.

Figure 10 shows the relative distances (from Quad 1 to Quad 2) estimated with the method proposed in Section 2. It can be seen that these measurements are good enough to be used to update the filter (see Section 4). It is important to remark that the observability results presented in Section 3 depend on these measurements. The lower-right plot of Figure 10 shows an image frame captured from the monocular camera carried by Quad 2. In this case, the projection of the landmarks can be appreciated, as well as the projections of the four rotors of Quad 1 needed to compute the homography.

In order to compare the quality of the measurements obtained with the fixed stereo system and those obtained with the cooperative pseudo-stereo system, some computer simulations were performed. In this case, the error was computed for the estimated landmarks’ positions, assuming that the position of Quad 1 was perfectly known along the flight trajectory. For the fixed stereo system, the camera-camera calibration is perfectly known. For the cooperative pseudo-stereo system, the camera-camera calibration is obtained from the homography, and therefore, it presents a certain level of error.

**Figure 11:**
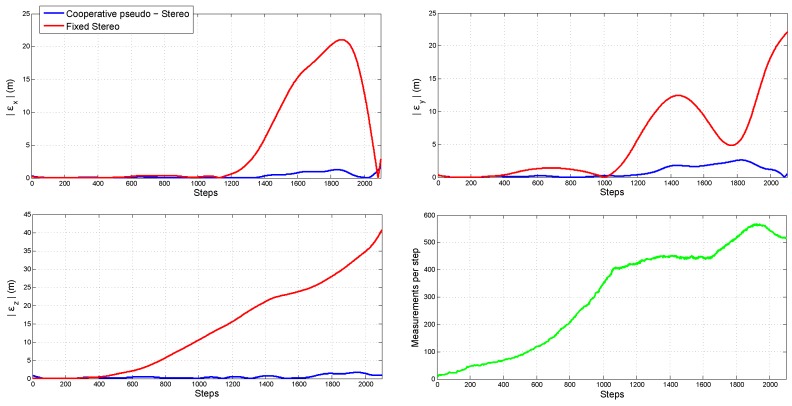
Comparison of the quality of the measurements obtained from a fixed stereo system and those obtained with the cooperative pseudo-stereo system.

Figure 11 shows the absolute value of the mean error obtained for both methods. In this experiment, the same measurements were performed for both systems. In the lower-right plot, the number of measurements per frame is shown. In the case of the fixed stereo system, the accuracy of its measurements is affected by the small baseline between cameras. This is especially notorious when the vehicle moves far away from the landmarks (the altitude is increased). In the case of the cooperative pseudo-stereo system, the error in estimation is much better bounded, although the calibration of the system is not perfectly known. A suitable explanation has to do with the possibility of having an arbitrarily greater baseline between the cameras.

**Figure 12:**
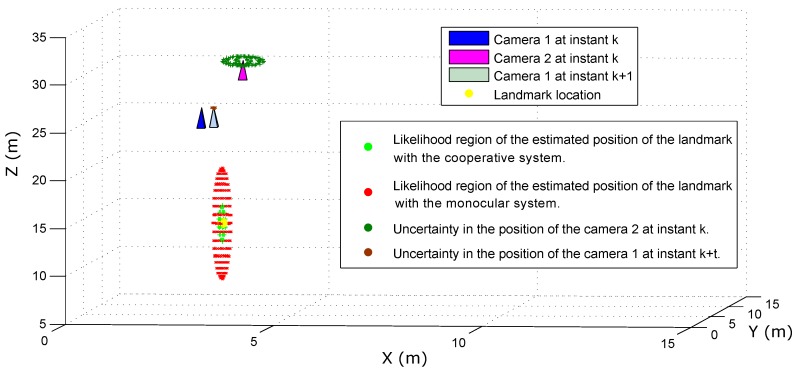
Measurement of a single landmark using: (i) cooperative pseudo-stereo system; and (ii) the delayed monocular initialization method.

Figure 12 illustrates the above fact. In this case, the statistical results obtained from simulating the measurement of a single landmark with (i) the cooperative pseudo-stereo system and (ii) a monocular method are presented. In the simulation, the UAV-Camera 1 system is located at [x,y,z]=[3,3,25] at instant *k*. The UAV-Camera 2 system is located at [x,y,z]=[4,3,30] at instant *k*. Thus, the baseline in the cooperative system is equal to 5.09 meters. A landmark is located at [x,y,z]=[3.5,3,15]. In order to model the inaccuracies associated with the cooperative pseudo-stereo approach, the estimated location of the UAV-Camera 2 system was modeled by adding a Gaussian noise with σ=50 cm to its actual location. In order to emulate the monocular measurements, it is assumed that the UAV-Camera 1 system was moved (at some instant k+t) to [x,y,z]=[3.3,3,25.1] to generate a parallax with respect to the landmark. Thus, the baseline in the monocular system is equal to 0.31 meters. The drift associated with the estimated displacement of the UAV-Camera 1 system is modeled by adding Gaussian noise with standard deviation σ=5 cm to the actual location at instant k+t. In all cases, the angular measurements provided by the cameras are corrupted by Gaussian noise with σ=3 degrees. Using the above conditions, a Monte Carlo simulation with 1000 executions has been used to estimate the landmark position with linear triangulation. In Figure 12, ellipsoids are used to illustrate the uncertainties in the estimated positions. According to the simulation results, it is better to have a larger baseline between two cameras with greater position uncertainty (like the cooperative system) than a small baseline with small uncertainty (like monocular measurements with low parallax).

In practical applications, there are several related factors that can severely also affect the performance of a system. For instance, in visual SLAM, the data association problem is critical for these approaches to be reliable. Although currently, there are several methods available for rejecting outliers, it is difficult to completely eliminate this problem. In addition, in cooperative visual systems, the data association problem can be extended from the single-image case to the multiple-image case. Furthermore, a problem that can arise in multi-robot systems, contrary to the mono-robot systems, is related to the communication issues between robots. This problem can cause loss of information or even make the interchange of information impossible during certain periods.

In order to take into account the above practical considerations, a set of additional computer simulations is presented. In this case, based on the same simulation setup used previously, the following aspects are now added: (i) outliers for the visual data association in each camera; (ii) outliers for the cooperative visual data association; (iii) outages of communication between robots; (iv) failures in the homography-based technique used for estimating the relative distance between robots.

In order to emulate the failures of the visual data association process, 5% of the total number of visual correspondences are forced to be outliers in a random manner. In this case, each outlier is modeled by means of a big measurement error of $\sqrt{e_{u}^{2}+e_{v}^{2}}=56\pm 14\sigma$ pixels. With the objective of having a good insight into the performance of the proposed method, under the above conditions, a comparison with a reliable general method is carried out. In this case, the method chosen is a monocular SLAM system aided by measurements of the position given by a GPS and attitude measurements obtained from an IMU (monocular SLAM + GPS + IMU).

Table 3 shows the number of failures introduced into the simulation: (i) the number of outliers introduced in the visual tracking process of Quad 1; (ii) the number of outliers introduced in the visual tracking process of Quad 2; (iii) the number of outliers introduced in the visual data association process used for cooperatively measuring the landmarks by means of Quad 1 and Quad 2; (iv) the number of outages in communication between robots, which result in filter update not being carried out with the information given by Quad 2; and (v) the number of failures in the homography-based technique, which result in the filter update not being carried out with the information given by the relative distance between the Quads.

**Figure 13:**
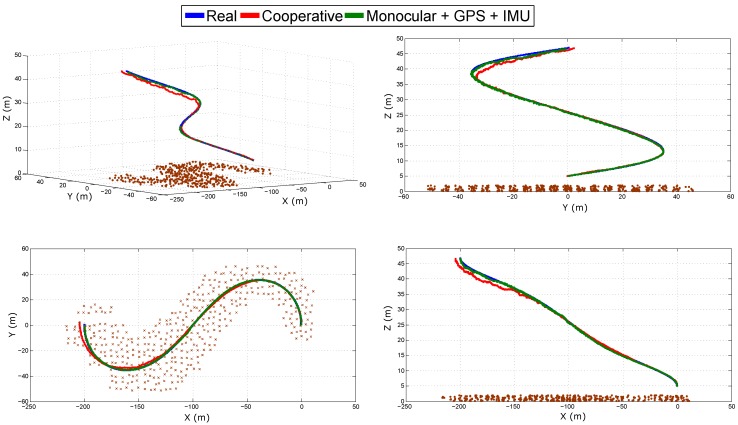
Estimated trajectory of Quad 1 obtained with the two configurations.

**Figure 14:**
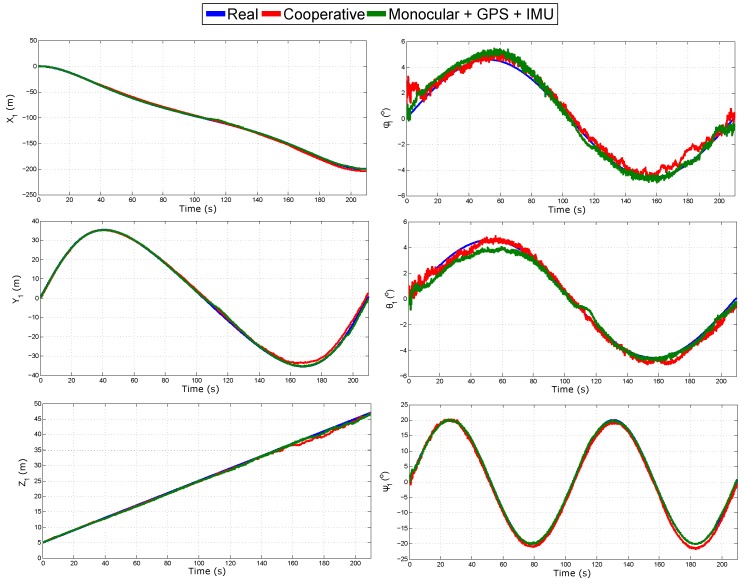
Estimated state of Quad 1.

Figure 13 shows the real and estimated trajectory obtained with the two configurations: (i) cooperative SLAM; and (ii) monocular SLAM + GPS + IMU. Figure 14 shows the evolution over time of the real and estimated states (position and orientation) of Quad 1 obtained with both configurations. Note that in this case, only the trajectory of Quad 1 is presented for illustration purposes, but estimates of Quad 2 are closely similar to those presented for Quad 1. Table 4 summarizes the mean squared error for the position in the three axes of Quad 1 obtained with both configurations. In this simulation, both configurations have a good performance, in the case of monocular SLAM + GPS + IMU, this result was expected, since this system has enough sources of information to determine all the states. The cooperative system shows a good performance despite all the failures introduced into the system. The above study provides a good insight about the robustness of the proposed (cooperative) system.

Table 5 provides an insight into the performance of the proposed method for estimating the features map. In this case, the total (sum of all) of the mean squared errors for the estimated position of the landmarks is presented for both configurations. Furthermore, the total of the mean squared errors for the initial estimated position of the landmarks is presented. Note that the results are presented for each coordinate of the reference frame *W*. The results show that the proposed cooperative system has a better performance than the monocular SLAM + GPS + IMU system, regarding the error obtained in the estimation of the position of the landmarks, although the latter has more sources of information provided by its sensors.

## 6. Conclusions

In this work, a vision-based cooperative SLAM system with application to unmanned aerial vehicles has been presented. The general idea is to take advantage of a cooperative UAV scheme in order to improve the accuracy and consistency of the state estimation process of the whole system. To achieve this purpose, it was proposed to add some relative distances between the robots as system measurements for updating the EKF. These measures provide metric information to the system, unlike other configurations where the scale of the system is a problem. Through a non-linear observability analysis, it is verified that the observability of the cooperative system improves the observability obtained for a single-robot configuration. In this case, the observability of the system is improved by adding the measures of relative distances. Sufficient conditions required for obtaining the observability results were established. In order to infer the 3D knowledge of the position of the landmarks for initializing the map features with less uncertainty, in the proposed method, pseudo-stereo systems are formed from pairs of aerial robots.

An extensive set of computer simulations was performed in order to validate the proposed method. In the computer simulations, the proposed system was compared against four single-robot configurations of visual SLAM. Based on the results of the simulations, it can be observed how the proposed method (cooperative) improves the estimation of the state with respect to the other configurations. The difference in the performance of the systems is especially notorious when the distance from the cameras to the landmarks increases. Furthermore, it was shown that the consistency of the filter is improved with the proposed method. Computer simulations also show that the accuracy of the measurements obtained from the pseudo-stereo system is better than the measurements obtained from a stereo system with a fixed small baseline.

In computer simulations, an effort has been made in order to emulate several aspects regarding applicability in real scenarios of the proposed approach. For instance, the data association problem has been considered by emulating outliers (mismatches) during the tracking of visual features on each monocular camera, as well as on the pseudo-stereo matching. Furthermore, issues for the multi-robot communication were considered, as well as failures on the homography technique used to provide measurements of the relative distance between robots. However, although computer simulations are useful for evaluating the full statistical consistency of the methods, they can still neglect important practical issues that appear when the methods are used in real scenarios. In this sense, it is important to note that future work should be focused on developing experiments with real data in order to validate the applicability of the proposed approach fully. Therefore, it should be interesting to investigate more practical aspects, like the homography-based technique or the pseudo-stereo matching process.

## Figures and Tables

**Table 1:** Results of the non-linear observability analysis.

	Unobservable Modes	Unobservable States	Observable States
Monocular	5	${x_{c}}^{j}$, ${v_{c}}^{j}$, ${x_{a}}^{i}$, $\psi_{c}^{j}$, ${\dot{\psi }}_{c}^{j}$	$\phi_{c}^{j}$, ${\dot{\phi }}_{c}^{j}$, $\theta_{c}^{j}$, ${\dot{\theta }}_{c}^{j}$
Cooperative	3	${x_{c}}^{j}$, ${x_{a}}^{i}$	${v_{c}}^{j}$, ${\lambda_{c}}^{j}$, ${\omega_{c}}^{j}$

**Table 2:** Mean squared error in the position estimation.

	${\mathrm{MSE}}_{x}$ (m)	${\mathrm{MSE}}_{y}$ (m)	${\mathrm{MSE}}_{z}$ (m)
Cooperative	0.36	0.05	0.008
Monocular	30.31	6.28	2.36
Stereo	28.42	5.33	3.82
Monocular + Stereo	12.64	1.51	2.85

**Table 3:** Number of failures introduced into the simulation.

	No. of Quad 1 Visual Outliers	No. of Quad 2 Visual Outliers	No. of Cooperative Visual Outliers	No. of Communication Outages	No. of Homography Failures
Cooperative	9002	8400	1706	210	420
Monocular + GPS + IMU	9535	-	-	-	-

**Table 4:** Mean squared error in the position estimation.

	${\mathrm{MSE}}_{x}$ (m)	${\mathrm{MSE}}_{y}$ (m)	${\mathrm{MSE}}_{z}$ (m)
Cooperative	0.85	0.23	0.07
Monocular + GPS + IMU	0.01	0.04	0.05

**Table 5:** Total mean squared error in: (i) the position estimation of the landmarks ($MSE_{xm}$, $MSE_{ym}$, $MSE_{zm}$); and (ii) the initial position estimation of the landmarks ($MSE_{xmi}$, $MSE_{ymi}$, $MSE_{zmi}$).

	${\mathrm{MSE}}_{\mathrm{xm}}$ (m)	${\mathrm{MSE}}_{\mathrm{ym}}$ (m)	${\mathrm{MSE}}_{\mathrm{zm}}$ (m)	${\mathrm{MSE}}_{\mathrm{xmi}}$ (m)	${\mathrm{MSE}}_{\mathrm{ymi}}$ (m)	${\mathrm{MSE}}_{\mathrm{zmi}}$ (m)
Cooperative	7.84	14.87	6.29	74.21	83.72	45.50
Monocular + GPS + IMU	21.80	30.31	13.27	394.05	427.91	193.99
